# Study of regulatory promoter polymorphism (-248 G>A) of Bax gene in patients with gastric cancer in the northern provinces of Iran 

**Published:** 2016

**Authors:** Seyedeh Habibeh Mirmajidi, Mojtaba Najafi, Seyedeh Tahereh Mirmajidi, Nafiseh Nasri Nasrabadi

**Affiliations:** 1 *College of Sciences****, ****Sari Agricultural Sciences and Natural Resources University, Sari, Iran*; 2 *Department of Animal Sciences and Fisheries, Sari Agricultural Sciences and Natural Resources University, Iran*; 3 *Tehran University of Medical Sciences, Cancer Institute, Tehran, Iran*; 4 *Student Research Committee, Mazandaran University of Medical Sciences, Sari, Iran *

**Keywords:** Promoter, Bax gene, Gastric cancer, Polymorphism, PCR-RFLP

## Abstract

**Aim:**

The aim of this study is to evaluate the polymorphism in Bax gene and its association with some clinical pathology traits in gastric cancer.

**Background:**

Gastric cancer is considered as the fourth most common cancer in the north and northwest of Iran. Bcl2 family has a key role in regulation of apoptosis, and any changes in the expression of Bcl2 lead to cancer.

**Patients and methods:**

Blood samples were collected from 100 cases and 89 controls in the northern provinces of Iran to evaluate promoter polymorphism (-248G<A) of Bax gene. Genotyping was carried out by PCR-RFLP method.

**Results:**

The result of this study demonstrated the existence of polymorphism in the above-mentioned region of Bax gene. Sixty-nine patients (%69) with genotype GG and 31 patients (%31) with genotype AG were observed in the case group. No mutant genotype was found among cases. Sixty-seven individuals (%75/28) with genotype GG, 21 individuals (%23/59) with genotype AG and only one mutant genotype (AA) were demonstrated in the control group. The bioinformatics analysis showed that this polymorphism removed the probable Sp1 motifs, which may affect its expression in the cells.

**Conclusion:**

Allele G was the most frequent between both patient and control samples. Polymorphism may be effective on Bax expression, but it requires further investigation. Our results showed significant effects between genotypes and features of gender and age, whereas no significant relation were observed between the genotypes and grade, stage as well as smoking traits.

## Introduction

 Gastric cancer is the second worldwide cancer disease for its mortality rate, after esophagus cancer (1). Due to its poor prognosis and its highly rated cause of death, it is known as the death captain (2). A drastic rise was observed in the number of cases and deaths caused by gastric cancer during the second half of the 20^th^ century. There is a geographic diversification in the occurrence of gastric cancer. Most cases are recorded in Japan, China, South America, and significantly less in Western Europe and the United States (3). Gastric cancer is more prevalent in men aged 40 and over. They face higher risks for this type of cancer (4). Statistics demonstrate an increasing rate of gastric cancer outbreak in the north provinces of Iran, especially in Mazandaran and Golestan provinces (5). In general, various factors are related to apoptosis, including bacteria, environment, age, gender, genetic factors, the implemented mutations in tumor’s suppressor genes and apoptosis related genes (6-9). Apoptosis is a natural process, which causes preservation of homeostasis in the natural tissues of the body by making a balance between cellular proliferation and death, as well as removing the damaged and old cells. Controlling apoptosis or any disorders in that regard have effective roles in the malignant deformation process, cancer progress and metastases (10, 11). Apoptosis occurs via two different pathways, including the death-receptor pathway and mitochondrial pathway (12). Bcl-2 family, as the most important regulator in the mitochondrial pathway, contains both anti-apoptotic proteins such as Bcl-2 and Bcl-xL, as well as pro-apoptotic proteins such as Bax, Bad and Bak (13-15). Bax gene is the first defined apoptotic gene that is directly activated by p53 and is located on human chromosome 19q13.3 with 6 exons (16, 17). Gene polymorphisms are known to be cancer risk detection parameters and a variety of genes, which are related to increased cell proliferation and lost of apoptosis, are being investigated worldwide. Similar to all other cancers, altered apoptosis regulation is one of the major problems in gastric cancer (18). This direct regulation puts a spotlight on Bax gene, especially for understanding the mechanism underlying the carcinogenesis process and possible therapeutic approaches. A promoter polymorphism G (−248) A in the 5’ UTR of Bax gene may alter the regulation of apoptosis in carcinogenesis (19).

So far, a few studies have been reported about polymorphism relationship between (-248G<A) position of Bax gene and cancer risk. Most of these studies were done on the blood cancer. They demonstrate polymorphism in this position, which is associated with decreasing of Bax expression, leading to increasing blood cancer (19). Polymorphisms of G-248A and its expression in the patients with blood cancer were evaluated in a study.by jane starczynski et al. Above mentioned polymorphism was observed in %23 of patients and %15 of normal groups but no significant relationship was observed in allele frequency between the patients and the control groups. The presence of these polymorphisms in patients with blood cancer has affected the therapy process and the overall survival of the patients (20). Polymorphisms in G-248A SNP were analyzed in another study, using PCR-RFLP technique, on the women with breast cancer in Turkey, including 53 patients and 82 women in the control group. The results did not statistically demonstrate any significant differences in genotype and allele frequency between control and case groups. Researchers report that the mentioned polymorphisms cannot change the sensitivity of individuals against breast cancer in Turkish women, and in general, this has been considered as the first report of polymorphism in the G-248A region of the Bax gene in the breast cancer (21). 

## Patients and Methods

**Patients and samples**

A total of 189 individuals (100 patients with gastric cancer and 89 normal subjects) in this experimental study were analyzed for Bax gene polymorphisms in north of Iran, during 2011-2014. Demographic data (age, sex) and utilization of risk factors (smoking) were extracted from the patient's files, or were collected by telephone interviews with the patients or their relatives. The clinicopathological parameters included clinical staging in accordance with the TNM classification of (AJCC/UICC) and grade. 

**DNA extraction and amplification**

Modified salting out method was used for DNA extraction from blood samples. 1One μl of genomic DNA (100 ng/ μl) was amplified in each 25 μl reaction, by 1.25 U EasyTaq DNA polymerase with 2 μl of 2.5 mM dNTPs and 0.5 μl of each primer (table 1). PCR conditions were set as follows: 95°C for 5 min, 30 cycles of 96°C for 30 s, 61°C for 30 s, and 72°C for 30 s, as well as a final extension step of 72°C for 7min. 

**Table 1:** Genotyping assays for the G-248A Bax gene polymorphism

Gene	Position	Primer sequences	PrPPoduct length	Enzyme	reference
BAX	-248G>A (rs4645878)	5- CATTAGAGCTGCGATTGGACCG-3 G 5- CTCCCTCGGGAGGTTTGGT-3	109 bp	MSP1	25

**Genotyping**

Polymerase chain reaction (PCR)-restriction-fragment length polymorphism was performed to investigate the A-248G Bax genotype. After PCR amplification, 8μl product PCR from each sample was digested by Msp1 enzyme for 16 hours at 37°C. Then, the products were separated on a 12% acryl amide gel and silver nitrate staining.

**Bioinformatics Analysis**

Due to the SNP existence in the promoter region, we evaluated the location of this SNP in a binding site. DNASIS MAX software was used in the evaluation, in this study.

**Statistical analysis**

POPGENE Ver.1.3 was used to determine the genotype frequency and to evaluate Hardy-Weinberg equilibrium. The allele frequency of Bax SNP-248 was tested using Exact Fisher Test by SAS Ver.9.1. The logistic regression model was carried out to analyze the association of Bax polymorphism between case/control groups and the clinicopathological characteristics such as age, sex, grade, stage and smoking. All statistical tests were considered significant with a level of p≤0.05. Odds ratios (OR) adjusted for age were calculated. All statistical analyses were carried out with SAS 9.1. 

## Results

**Demographic data**

**Figure1:**
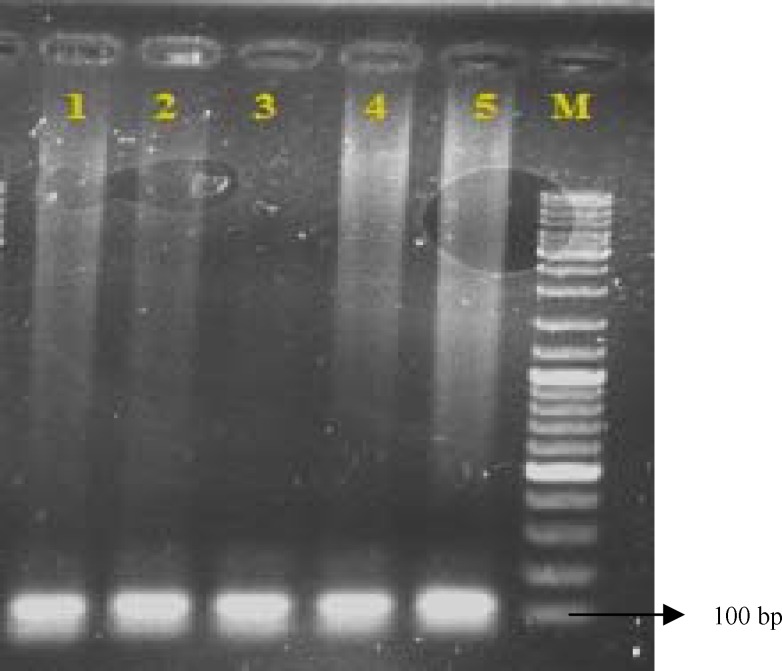
Products PCR obtained from 109 bp fragment in the promoter region of the Bax gene

The study group consisted of 74 males and 26 females giving a male/female ratio of 2.84. The age of the patients ranged from 21 to 88, with a median age of 62.67 years. Among the 89 individuals in the control group, 37 were male. The median age was 46.46 (range 27 –94) years. Most of them were poorly differentiated (73.97%). Bax gene, including 109 bp, was amplified by the specific primers from human genomic DNA, as expected (Figure 1).

**Genotyping by PBR**

**Figure 2:**
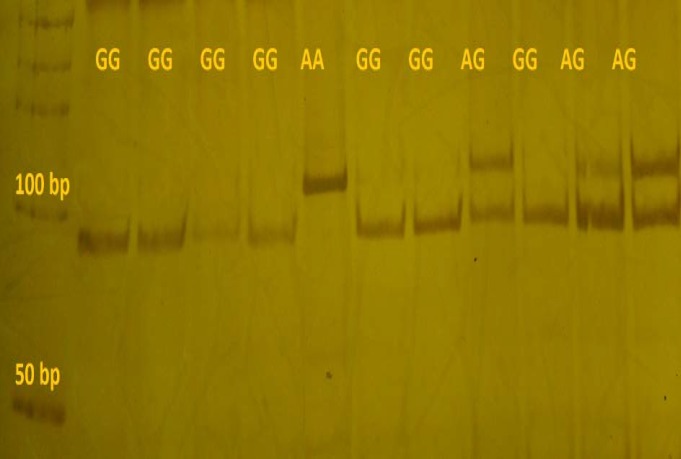
Samples of genotypes in Bax gene using PCR-RFLP

*MspI* enzyme was used for genotyping, from which it produced two fragments of 20 and 89 bp, obtained after digestion in GG genotype. AG genotype produced three bands (20, 89 and 109 bp) and AA genotype, which lacks the restriction site, and produced a 109bp fragment (Fig2). The genotype frequency of Bax (-248 G>A) in case samples (patients) showed 66.66% GG and 33.33% AG, but AA genotype was not observed in this study. Also, the normal subjects had an allelic frequency of 75.28% for GG, 24.69% AG, and 1.12% AA.

G-allele frequency in patients affected by gastric cancer was 0.88, and 0.84 in the control group. A-allele frequency in patients with gastric cancer was 0.11, while it was 0.15 in the control group.

After the evaluation of obtained genotypes, SNP was evaluated using DNASIS MAX software. Our results showed this SNP is located in a probable binding site. The bioinformatics analysis showed that this polymorphism removed the probable Sp1 motifs in AA genotype, which may affect its gene expression in cells.

The genotype frequency of the mentioned SNP was tested using Popgene Ver.1.3, for Hardy-Weinberg equilibrium (HWE). The chi-square test was used to determine whether the subjects met Hardy-Weinberg equilibrium or not. We confirmed that both case and control groups, were not compatible with the HWE (p< 0.05). The comparative allele frequency showed that there was no significant differences in both case and control groups (p>0.05). The logistic regression model was carried out to analyze the distribution of Bax -248SNP polymorphism between the case and control groups and the clinicopathological characteristics of gastric cancer. (The results of which are shown in table 2). Moreover, there is no relation between genotypes and clinicopathological characteristics (Table 3). 

**Table 2:** Bax (-248G > A) genotype distribution in patients and the control group

	**Control (n=89)**	**Case (n=100)**	**P value**	**OR** *
GG	67(75.28%)	69 (69%)		1.00
AG	21(23.59%)	31 (31%)	0.28	0.686
AA	1 (1.12%)	0		
All GA	0/84 0/15	0/88 0/11	0.77	0.883

* Logistic regression model, adjusted for diagnostic age

## Discussion

The event of apoptosis leads to the death of cells without releasing detrimental substances into the tissue. As a member of the Bcl-2 family, Bcl-2-associated X Protein (Bax gene) acts as an important regulator of apoptosis. With respect to the important roles of Bax in apoptosis, it is biologically a reasonable idea that its polymorphism may have a great role in the risk of cancer. (22)

The sensitivity of cells to apoptotic stimuli may depend on the balance of pro- and anti-apoptotic bcl2 proteins (22). Cells are more sensitive to apoptosis, when they are exposed to an excess of pro-apoptotic proteins; therefore, the cells will tend to be more resistant. The pro-apoptotic bcl-2 proteins are the sensors for cellular damage or stress in cytosol. They relocate to the surface of the mitochondria, when they are exposed to stress conditions. The interaction between pro- and anti-apoptotic proteins obstructs the normal function of the anti-apoptotic bcl-2 proteins, and can create pores in the mitochondria, finally leading to the release of cytochrome C and other pro-apoptotic molecules from the intermembrane space (23, 24). Bax promoter contains response elements for an important tumor suppressor p53 and this affects the gene expression (16). TP53 regulates cellular apoptosis, via direct binding to the promoter elements and activating transcription of some apoptosis-regulatory genes, such as Bax (25, 26). Polymorphisms (SNPs) in Bax promoter may involve the process of carcinogenesis by altering its own expression and the cancer related genes (27).

The changed expression of Bax protein is presumably relevant to carcinogenesis. However mutations that result in deregulation and correlations with cancer are of the interests of researchers, since it has relationships with different types of cancer diseases (table.4). 

The aim of this study was to evaluate whether polymorphisms of G-248A in the Bax gene was associated with the risk of developing gastric cancer. Our results demonstrated that the - 248AG polymorphism removed a Sp1 binding site of promoter in AA genotype, which may affect its gene expression. 

The expression of the pro-apoptotic protein Bax appears to be regulated through p53-dependent transcriptional activation in human cells (28). The presence of Sp1 or a Sp1-like factor through a 6 base pair motif (5'-GGGCGT-3') is necessary for transcriptional regulation of Bax by p53 (29). Sp1 is an important motif that has an important role in the transcription of many genes, which contain GC boxes in their promoter (30). Sp1 protein comprises of several domains, of which the DNA binding domain is the most conserved among Sp family. Sp1 could bind GC-rich elements, which are common regulatory elements in numerous gene promoters (31). Also, Sp1 is considered as a vital transcription factor for many genes due to regulation of multiple functions, including tumor cell survival, growth, angiogenesis, abnormal Sp1 expression. Moreover, its activation may contribute to gastric cancer development (32). 

In an association study between polymorphisms of - 938C/A and Thr43Ala in the BCL-2 gene and G - 248A in the BAX gene and the risk of developing non-Hodgkin lymphoma (NHL), it was reported that the - 248AG + AA genotype of the BAX gene might be susceptible genotypes for NHL (33).

As a transcriptional factor, tumor protein 53 (TP53) regulates the expression of the BAX and Bcl2 genes. A study was done to evaluate the two polymorphisms of BAX (-248 G>A) and BCL2 (-838C>A) in promoter regions, as well as the functional polymorphism of TP53 (Arg72Pro) and their associations with the risk of SCCHN in a US non-Hispanic white population. The results showed that TP53 heterozygous and BAX homozygous variant genotype (AA) lead to a significant increase in the risk of SCCHN, as compared to BAX homozygous genotype (GG) or combined genotypes (GG + AG). One of the probable reasons of that may be due to the existence of weakening interaction between the TP53 heterozygous and -248 AA genotype in the cells (19). 

Another study was performed to revalidate the association of Bax G-248A polymorphism with lung cancer risk in Chinese population. The researchers reported that Bax -248A allele had significantly higher transcriptional activity as compared to G allele, and other alleles, Bax-248A allele is significantly associated with decreasing lung cancer susceptibility in Chinese population (34). In addition, the results showed significance in gene-smoking interaction between Bax G-248A polymorphism and the smoking trait existing among the light smokers, whereas in our study no association was observed between this polymorphism and gastric cancer. Furthermore, Chen et al. (2007) reported no association between this polymorphism and squamous cell carcinoma of the head and neck (SCCHN). The comparative analysis of the frequencies of polymorphic variants of locus‐248G> A of BAX gene in chronic lymphocytic leukemia (CLL) patients revealed a significant difference in GG‐genotype among the CLL patients and the control group. Thus, as a result, the GG‐ genotype and G‐allele of the polymorphic locus‐248G>A of BAX gene have been identified as risk markers for CLL, which is not consistent with other studies. Since other studies showed that AA genotype may lead to a significant increase in cancer risk (35). Seventy-six patients in this study were male and 24 were female, and the median age was 3.16: 1 (range from 21 to 88 years). Also, most of them were poorly differentiated (73.97%). Hajian et al (2006) indicated that male to female ratio was 2.6:1. The mean age was 60.6 years and 14% of the patients were younger than 40 years of age when most patients are in advanced stages, which favors a poor overall survival (36). Based on gastric cancer’s specific feature in Iran, the patients are diagnosed at advanced stages (III, IV) (37), most of whom perfectly corresponded with the results of this study. The incidence of gastric cancer increases with age and it doubles through each decade in life: 55 to 65 and 65 to 75 of age and above (38). In this study, 76 and 24 percent of patients were men and women, respectively, which is in conformity with the findings by Zali et al. (39) and Biglerian et al. (40). 

Yildiz et al. showed that Bax-248GA genotype and allele frequencies between the control group and breast cancer patients were not statistically significant (p = 0.866, p = 0.856 respectively) (41). Generally, the results about the relation between Bax-248GA polymorphism and cancer risk are complicated.

We did not observe a statistically significant difference in Bax G(−248)A genotype and allele frequencies between control and gastric cancer patients. However, we observed that demographic characteristics such as age and gender are related to specific genotypes. In any case, no correlation was observed between clinicopathological data (stage, grade) and smoking with specific genotypes.

**Table 3:** Relationship between Bax (-248G > A) polymorphism and known clinicopathological variables

**Clinicopathological Variables**		**Genotypes**	**Adjusted**		
	All	**AG**	**P-value**	**GG**	**OR**
Age					
≥ 45	11	2		9	1.00
> 45	89	29	0.0290*	60	2.255
Gender					
Male	76	21		55	1.00
Female	24	8	0.0001**	16	12.341
Habit					
Cigarette smokers	23	9		14	1.00
Non-smokers	45	16	0.8117	29	0.836
Stage					
I, II	19	8		11	1.00
III, IV	54	19	0.7867	35	0.731
Tumor Grade					
I, II	24	13		11	1.00
III	24	11	0.5642	13	1.397

All statistical tests were two-sided with a significance level of p≤0.05.

* Logistic regression model, adjusted for diagnostic age. NTM Stage: I, II, III, IV

However, it does not indicate the lack of significant difference among other populations in Iran and other cancer cases. This genotype may have no roles in increasing the risk of gastric cancer in the people under consideration.

One probable reason for the insignificance association between the obtained genotypes and clinicopathological data, such as grade, stage and smoking could be the few number of samples. Further analysis and research will be required among other populations, for more precise and irrevocable results. On the other hand, other regions of Bax gene may interfere in gastric cancer. It is suggested for future investigations on the whole gene by high throughput SNP chip or direct sequencing (NGS platforms). 

Due to their jobs exposed mainly to carcinogen materials, chemical material and X ray, as well as consuming alcohol and cigarette more than women, men are more susceptible to cancer diseases (15). However, factors involved in cancer incidence require more investigation. 

This paper represents the first study on Bax G (−245) a polymorphism in patients with gastric cancer in the northern provinces of Iran and gives rise to the possible effects of this polymorphism in gastric cancer progression. However, further confirmations in more extensive studies are needed to clarify the role of G (−248) a polymorphism in gastric cancer.

Allele G was the most frequent among the patients and individuals in the control group. Due to removing of Sp1 motif, polymorphism may have an effect on Bax expression, but it needs further investigation. We did not find a correlation among the polymorphism of A-248G Bax gene and gastric cancer risk or between this polymorphism and stage, grade, and smoking. However, our results showed significant effects between genotypes and gender and age.

Moreover, in order to have a better and comprehensive understanding of the relation between this polymorphism and vulnerability to cancer, it would be appropriate to extend the investigation to a wider range of human populations with different types of cancer.
